# A stress management intervention for adults living with HIV in Nigerian community settings: An effects study: Erratum

**DOI:** 10.1097/MD.0000000000013504

**Published:** 2018-11-21

In the article, “A stress management intervention for adults living with HIV in Nigerian community settings: An effects study”,^[1]^ which appeared in Volume 97, Issue 44 of *Medicine*, Dr. Kenneth Chukwuemeka Obetta's name appears incorrectly as Obetta Emeka.
